# Recurrent Pleural Effusion and Grade 3 Pyrophosphate Uptake in Cardiac Immunoglobulin Light Chain (AL) Amyloidosis: A Transthyretin Amyloidosis (ATTR) Phenocopy

**DOI:** 10.7759/cureus.112551

**Published:** 2026-07-12

**Authors:** Amanda Carreon Simental, David Villanueva-Lechuga, Leonor Bonilla Quezada, Jose Aldo Sanchez Aguirre, Carolina Martinez-Mijares

**Affiliations:** 1 Internal Medicine, Centro Medico Nacional 20 de Noviembre, Mexico City, MEX; 2 Internal Medicine, National Autonomus University of Mexico, Mexico City, MEX; 3 Pathology and Laboratory Medicine, Centro Medico Nacional 20 de Noviembre, Mexico City, MEX

**Keywords:** al cardiac amyloidosis, cardiomyopathy, diastolic heart failure, echocardiography, nuclear medicine, preserved ejection fraction, restrictive

## Abstract

Cardiac immunoglobulin light chain (AL) amyloidosis classically presents with biventricular heart failure and increased wall thickness. Recurrent unilateral pleural effusion as the inaugural manifestation is rarely recognized, and grade 3 99mTc-pyrophosphate (PYP) uptake is conventionally attributed to transthyretin amyloidosis (ATTR) amyloidosis. We describe a 66-year-old woman with treatment-resistant heart failure with preserved ejection fraction who presented with refractory pleural effusion requiring three large-volume thoracenteses. Echocardiography showed concentric hypertrophy with apical-sparing strain, and PYP scintigraphy demonstrated grade 3 myocardial uptake. Serum free light chains revealed a markedly elevated lambda fraction (33,562 mg/L) with a profoundly suppressed kappa-to-lambda ratio (0.001), and bone marrow biopsy confirmed coexistent multiple myeloma. First-line plasma cell-directed chemotherapy was initiated, but the patient died of septic shock and multi-organ failure 60 days after admission. This case illustrates two underrecognized phenomena: refractory unilateral pleural effusion as an inaugural feature of AL amyloidosis, and PYP-positive scintigraphy mimicking ATTR despite biochemically confirmed AL disease. Plasma cell dyscrasia must be excluded before any PYP-based diagnosis of ATTR amyloidosis is accepted, and refractory unilateral pleural effusion in a patient with diuretic-resistant heart failure warrants systematic evaluation for cardiac amyloidosis.

## Introduction

Immunoglobulin light-chain (AL) amyloidosis is a clonal plasma cell disorder in which misfolded light chains deposit in vital organs; transthyretin amyloidosis (ATTR) results from deposition of misfolded transthyretin protein, either due to hereditary mutations or age-related wild-type fibril instability. Although both subtypes produce an identical infiltrative cardiomyopathy phenotype, their treatments differ fundamentally: AL amyloidosis requires urgent plasma cell-directed chemotherapy, whereas ATTR is treated with transthyretin stabilizers, such as tafamidis - making accurate subtype identification a direct determinant of survival [1,2]. Cardiac involvement is the principal determinant of prognosis in AL amyloidosis [1,2]. Recurrent unilateral pleural effusion is an underrecognized inaugural manifestation of the disease and is frequently misattributed to right-sided heart failure rather than to direct pleural amyloid deposition [3].

A separate diagnostic challenge has emerged with the widespread adoption of bone-tracer scintigraphy. 99mTc-pyrophosphate (PYP) scintigraphy detects myocardial radiotracer uptake, graded on the Perugini scale from 0 to 3 based on the intensity of cardiac signal relative to bone; grade 2 or 3 uptake carries a positive predictive value exceeding 99% for ATTR amyloidosis and is now used as a non-biopsy diagnostic criterion - but only when a monoclonal protein has been formally excluded by negative serum and urine immunofixation electrophoresis and a normal serum free light chain ratio [4]. This distinction is critical: when a monoclonal protein is present, non-invasive imaging alone cannot reliably distinguish ATTR from AL cardiac amyloidosis, because both subtypes may coexist and their scintigraphic appearances are indistinguishable. In this setting, current guidelines recommend histological or proteomic amyloid typing by endomyocardial biopsy [4]. We report a patient in whom both diagnostic pitfalls converged: refractory unilateral pleural effusion as the presenting feature, and grade 3 PYP uptake in a patient ultimately found to have biochemically and histologically confirmed AL amyloidosis with coexistent multiple myeloma.

## Case presentation

The following case unfolded over approximately nine months. The patient first developed dyspnea and recurrent right-sided pleural effusion in January 2025, requiring two large-volume thoracenteses at outside facilities (January and March 2025). She was referred to our center in July 2025, at which time a third thoracentesis was performed on admission. Cardiac and hematologic evaluation was completed between July and August 2025, confirming the diagnosis of AL cardiac amyloidosis with coexistent multiple myeloma. First-line chemotherapy (CyBorD) was initiated on Day 1 (Cycle 1 Week 1) and Day 8 (Cycle 1 Week 2). The patient developed empyema and progressive multi-organ failure and died 60 days after admission and 23 days after the first dose of chemotherapy.

History and Physical Examination

A 66-year-old woman was referred in July 2025 for refractory right-sided pleural effusion and progressive dyspnea. Over six months, she had developed worsening dyspnea (New York Heart Association (NYHA) functional classes II-IV) [5], bilateral lower-extremity edema, and dysphonia. Three large-volume thoracenteses had been performed at outside facilities (2,500 mL and 1,500 mL within the previous four months, and 1,700 mL on the day of admission), with prompt re-accumulation despite optimization of loop diuretic therapy. An involuntary 20 kg weight loss over 12 months was reported. During transport, she became severely hypoxemic (oxygen saturation of 75% on supplemental oxygen).

On arrival, the patient was alert and in moderate respiratory distress: blood pressure of 90/60 mmHg, heart rate of 92 beats per minute, and oxygen saturation of 75% on a 3 L/min nasal cannula. There were decreased breath sounds and dullness over the right hemithorax, a regular cardiac rhythm without murmurs, elevated jugular venous pressure, and bilateral lower-extremity pitting edema. No macroglossia, periorbital purpura, or carpal-tunnel scars were identified. Heart failure with preserved ejection fraction had been diagnosed eight months earlier (November 2024) at a regional hospital and attributed empirically to long-standing hypertension. Comorbidities included primary hypothyroidism on stable levothyroxine and 30 years of systemic arterial hypertension; antihypertensive therapy had been spontaneously discontinued three months before admission because of progressive hypotension. There was no family history of cardiomyopathy or amyloidosis.

Differential Diagnosis

The combination of recurrent unilateral pleural effusion in a normotensive elderly woman with diuretic-resistant heart failure prompted a structured work-up. Infectious etiologies were considered unlikely given the absence of fever, leukocytosis, or elevated procalcitonin and sterile pleural cultures (Table 1). Malignant effusion and mesothelioma were excluded by lymphocyte-predominant transudative cytochemistry and repeated negative cytology. A connective-tissue disease screen revealed a positive antinuclear antibody (titer 1:80, homogeneous pattern), negative anti-Sm and anti-RNP antibodies, and low complement C3, a profile not meeting the 2019 European Alliance of Associations for Rheumatology/American College of Rheumatology (EULAR/ACR 2019) criteria for systemic lupus erythematosus [6]. Electrocardiography demonstrated low-voltage QRS complexes; 2D echocardiography showed concentric hypertrophy and apical-sparing strain that shifted the differential decisively toward an infiltrative cardiomyopathy, with cardiac amyloidosis as the leading hypothesis.

**Table 1:** Laboratory and pleural fluid findings on admission. NT-proBNP: N-terminal pro-B-type natriuretic peptide; LDH: lactate dehydrogenase. Reference ranges represent standard adult laboratory values.

Parameter	Result	Reference range
White blood cell count	5.2 x 10⁹/L	4.5-11.0 x 10^9^/L
Procalcitonin	0.07 ng/mL	< 0.05 ng/mL
NT-proBNP	30,378 pg/mL	< 125 pg/mL
Troponin I	165.6 ng/L	< 26 ng/L (assay-dependent, high-sensitivity)
Serum creatinine	1.4 mg/dL	0.6-1.1 mg/dL (female)
Beta-2-microglobulin	1.32 mg/L	0.8-2.2 mg/L
Urine Bence Jones protein	Positive	Negative
Pleural fluid total protein	2.85 g/dL	Transudate: pleural/serum protein ratio < 0.5 (serum total protein: 5.7 g/dL; ratio: 0.50)
Pleural fluid lactate dehydrogenase (LDH)	94 U/L	Transudate: pleural/serum LDH ratio < 0.6 and pleural LDH < 2/3 upper normal limit (serum LDH: 189 U/L; pleural/serum LDH ratio: 0.50)
Pleural fluid lymphocytes	95%	Lymphocyte-predominant: > 50%
Pleural fluid microbiology	No growth	No growth (sterile)
Pleural fluid cytology	Negative for malignant cells	Negative

Investigations

Selected laboratory and pleural fluid results are summarized in Table 1. The chest radiograph showed a large right pleural effusion with complete blunting of the costophrenic angle and a small left pleural effusion.

Twelve-lead electrocardiography (Figure 1) demonstrated sinus rhythm, a narrow QRS complex, left anterior fascicular block, low voltage in both limb and precordial leads, and a pseudo-infarction pattern. Despite echocardiographic concentric hypertrophy, the Sokolow-Lyon, Cornell, and Peguero-Lo Presti voltage indices remained below conventional hypertrophy thresholds [7-9], illustrating the classic voltage-to-mass discrepancy of cardiac amyloidosis.

**Figure 1:**
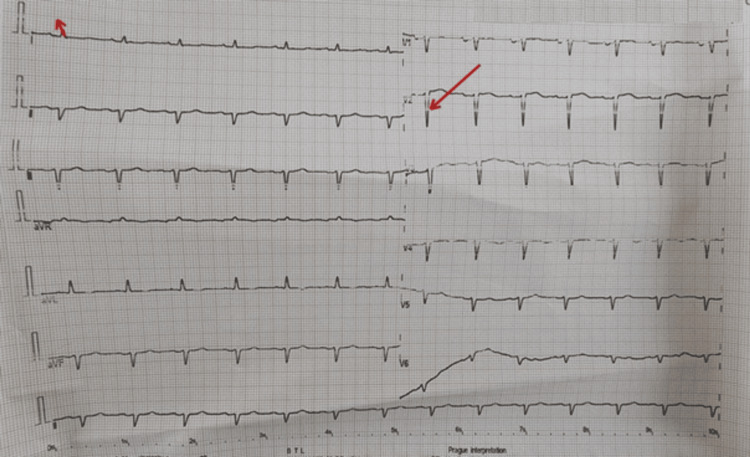
Twelve-lead electrocardiogram on admission. The red arrow (right panel) highlights the deep QS complex in lead V2, part of the pseudo-infarction pattern in the anteroseptal leads. The red arrow (top left) highlights low-voltage limb-lead complexes. Despite echocardiographic concentric hypertrophy, voltage indices remained below conventional thresholds, illustrating the classic voltage-to-mass discrepancy of cardiac amyloidosis.

The chest radiograph (Figure 2) showed a large right pleural effusion with complete blunting of the costophrenic angle.

**Figure 2:**
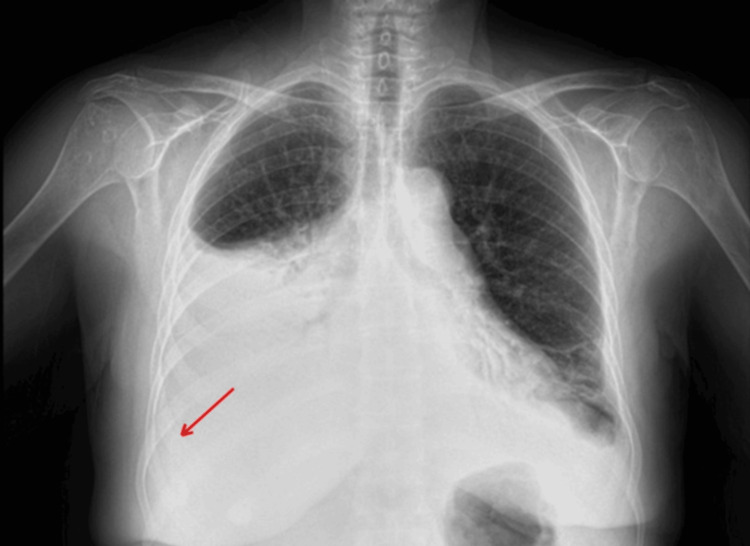
Posteroanterior chest radiograph on admission. The red arrow highlights the homogeneous opacification of the right hemithorax with complete costophrenic angle blunting and hemidiaphragmatic silhouette obliteration, consistent with a large pleural effusion; a smaller left basal effusion is also present.

Transthoracic echocardiography (Figure 3) showed concentric hypertrophy with an interventricular septum of 15.3 mm and a hyperrefringent, granular myocardial appearance, a biplane left ventricular ejection fraction of 57%, and grade 3 restrictive diastolic dysfunction (E/e': 40.2; estimated pulmonary capillary wedge pressure: 51.8 mmHg). Bull's-eye strain mapping demonstrated a global longitudinal strain of -17.9% with relative apical sparing (basal anteroseptum of -5.2%, basal inferolateral of -3.4%, contrasting with apical segments of -10.1% to -16.7%).

**Figure 3:**
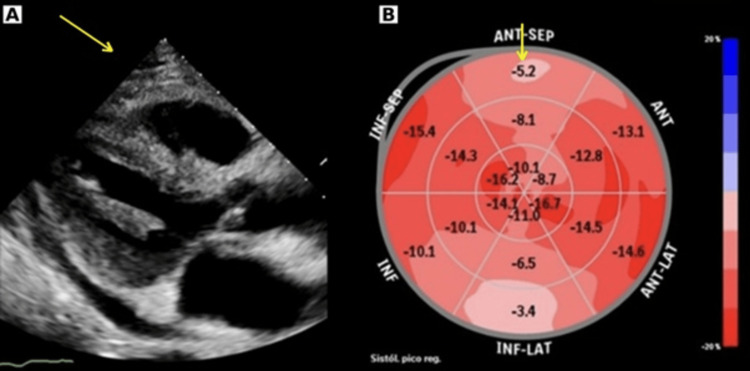
Transthoracic echocardiography. (A) Parasternal long-axis view; the arrow highlights the hyperrefringent, granular thickened septal wall, the classic sparkling appearance of amyloid infiltration. (B) Bull's-eye longitudinal strain map; the arrow highlights the basal anteroseptal segment, the most affected region of the relative apical-sparing pattern characteristic of cardiac amyloidosis.

Bone scintigraphy with PYP (Figure 4) demonstrated intense myocardial radiotracer accumulation with a heart-to-contralateral lung ratio of 2.60, exceeding paravertebral and approaching sternal bone uptake (Perugini grade 3) [10].

**Figure 4:**
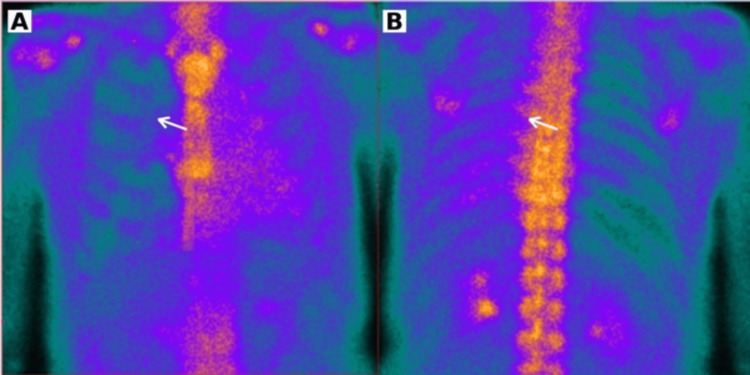
99mTc-Pyrophosphate scintigraphy at three hours after injection. (A) Anterior projection; the arrow highlights intense myocardial radiotracer accumulation. (B) Posterior projection; the arrow highlights corresponding diffuse myocardial and rib uptake. This pattern demonstrates radiotracer uptake greater than that of the ribs and paravertebral bone (Perugini grade 3; heart-to-contralateral lung ratio, 2.60). Although classically associated with ATTR amyloidosis, this scintigraphic pattern coexisted with a lambda-restricted plasma cell clone, illustrating an ATTR phenocopy that constitutes the core message of this report.

Per current diagnostic algorithms, hematologic screening was completed in parallel with cardiac imaging [4,11]. Urine protein electrophoresis (Figure 5) showed a discrete monoclonal spike within the beta-fraction (35.9% of total urinary protein, 10.8 mg/dL). Serum free light chain assay revealed free kappa 40.7 mg/L (reference: 3.3-19.4 mg/L), free lambda 33,561.8 mg/L (reference: 5.7-26.3 mg/L, more than 1,270-fold the upper reference limit), and a kappa-to-lambda ratio of 0.001 (reference: 0.26-1.65), unequivocally establishing a lambda-restricted plasma cell clone. Notably, electrophoresis was performed exclusively on urine, whereas free light chain quantification was restricted to serum. Serum immunoglobulins were uniformly suppressed (IgG: 478, IgA: 33, IgM: 24 mg/dL). Periumbilical fat-pad biopsy was Congo-red negative, a recognized limitation given the assay's variable sensitivity of 60-80% [2].

**Figure 5:**
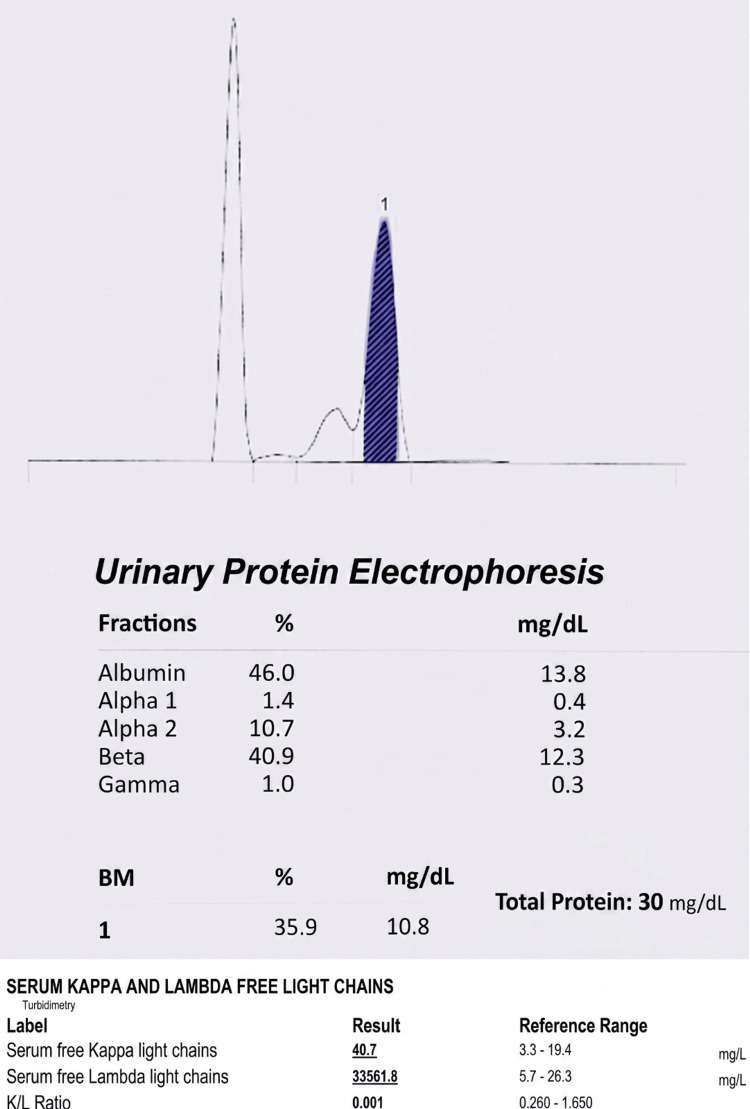
Urine protein densitometry curve and serum free light chain assay. Urine protein densitometry curve (upper panel) and serum free light chain quantification (lower panel). The arrow highlights the discrete monoclonal spike (band 1, 35.9% of total urinary protein, 10.8 mg/dL) within the beta fraction, distinct from the larger albumin peak. Serum free light chain quantification by turbidimetry revealed free lambda 33,561.8 mg/L (reference: 5.7-26.3 mg/L), free kappa 40.7 mg/L (reference: 3.3-19.4 mg/L), and a kappa-to-lambda ratio of 0.001 (reference 0.26-1.65) - a more than 1,270-fold elevation of lambda above the upper reference limit, unequivocally establishing a lambda-restricted plasma cell clone beyond reasonable diagnostic doubt.

Bone marrow biopsy with hematoxylin-eosin staining (Figure 6A) demonstrated 23% interstitial plasma cell infiltration, and CD138 immunohistochemistry (Figure 6B) confirmed expression in approximately 50% of marrow cellularity, fulfilling the International Myeloma Working Group criteria for multiple myeloma [12].

**Figure 6:**
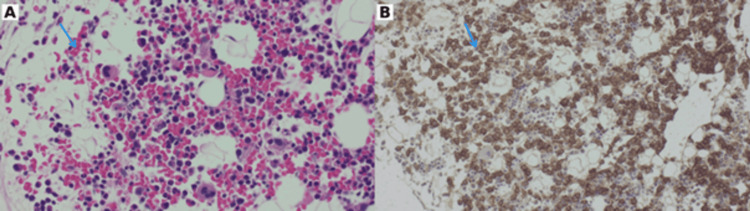
Bone marrow biopsy. (A) Hematoxylin-eosin stain (high power); the arrow highlights plasma cells with characteristic eccentric nuclei, a perinuclear hof, and clock-face chromatin, comprising 23% of nucleated marrow cellularity by morphologic count. (B) CD138 immunohistochemistry; the arrow highlights membranous and cytoplasmic staining in neoplastic plasma cells, representing approximately 50% of marrow cellularity. The apparent discrepancy between the morphologic estimate (23%) and the CD138 immunohistochemical result (50%) reflects the greater sensitivity of CD138 for detecting plasma cells that may be difficult to identify on routine hematoxylin-eosin staining because of atypical morphology, small cell size, or a dispersed interstitial distribution. Together, both estimates fulfill the International Myeloma Working Group criteria for multiple myeloma.

Diagnosis and Management

The final diagnosis was lambda-restricted AL cardiac amyloidosis with coexistent multiple myeloma, European 2015 modification of Mayo 2004 stage IIIb, and Mayo 2012 stage IV [13,14]. The grade 3 PYP scintigraphy in this biochemically confirmed AL setting represented an ATTR-mimicking phenocopy that did not modify the etiologic diagnosis once monoclonal disease was demonstrated.

First-line plasma cell-directed therapy was initiated on treatment Day 1 with the institutional cyclophosphamide-bortezomib-dexamethasone (CyBorD) protocol, consistent with regimens recommended by the Mexican Position Paper on Cardiac Amyloidosis [11]. Cycle 1 Week 1 (Day 1) and Week 2 (Day 8) each consisted of bortezomib 1.8 mg/m^2^ subcutaneously, cyclophosphamide 430 mg/m^2^ orally, and dexamethasone 40 mg intravenously. Daratumumab-based induction (the ANDROMEDA regimen) was preferable given the patient's Mayo IIIb/IV status [2], but was not logistically available. Throughout admission, the patient required continuous pleural drainage; an iatrogenic hydropneumothorax during catheter manipulation was managed with repositioning and underwater-seal suction.

Outcome and Follow-Up

The clinical trajectory after the second cycle was defined by the inherent fragility of advanced AL amyloidosis compounded by treatment-related immunosuppression. Right-sided pleural drainage transitioned to frankly purulent fluid (53,240 leukocytes/mm^3^, 100% neutrophils); cultures grew methicillin-susceptible *Staphylococcus aureus* and *Candida albicans*, and broad-spectrum antimicrobials were initiated. Acute-on-chronic kidney injury with hyperkalemia required renal replacement therapy, and atrial fibrillation with rapid ventricular response was rate-controlled with intravenous amiodarone within 48 hours. With progressive multi-organ failure, the family signed an advance directive declining resuscitation and invasive ventilation. The patient died 60 days after admission and 23 days after the first dose of CyBorD.

## Discussion

This case illustrates two underrecognized but clinically decisive phenomena: refractory unilateral pleural effusion as the dominant and inaugural manifestation of cardiac AL amyloidosis, and grade 3 99mTc-pyrophosphate uptake, conventionally regarded as virtually pathognomonic of ATTR amyloidosis, occurring in biochemically proven AL disease [1,2]. We acknowledge that current guidelines recommend histological or proteomic amyloid typing - ideally by endomyocardial biopsy - when grade 2 or 3 bone-tracer uptake coexists with a detectable monoclonal protein, given the possibility of concomitant ATTR and AL cardiac amyloidosis [3,15]. In our case, endomyocardial biopsy was not performed due to institutional limitations and the patient's hemodynamic instability; however, the magnitude of lambda free light chain elevation (33,562 mg/L, more than 1,270-fold above the upper reference limit) and the bone marrow findings fulfilling the International Myeloma Working Group criteria for multiple myeloma rendered the AL etiology overwhelmingly likely as the dominant driver of cardiac disease.

Pleural effusion is reported in approximately 1-6% of patients with systemic AL amyloidosis as a large refractory effusion, rising to 10-15% when smaller effusions are considered [3,16]. Although traditionally attributed to right-sided heart failure, a substantial proportion in fact reflects direct pleural amyloid deposition, producing persistently unilateral, transudative, diuretic-resistant accumulation [3,17]. Pleural involvement may itself be the sentinel event leading to systemic diagnosis [16,17], as in our patient, whose six-month history of recurrent right-sided effusion preceded any formal cardiac amyloidosis evaluation.

The most instructive element is the diagnostic discordance between PYP scintigraphy and the underlying amyloid subtype. The Gillmore non-biopsy diagnostic algorithm confers a positive predictive value exceeding 99% for ATTR when grade 2 or 3 myocardial uptake coexists with the absence of a monoclonal protein - defined by negative serum and urine immunofixation electrophoresis and a normal serum free light chain ratio [4]. Bone-tracer myocardial uptake has been documented in up to 40% of patients with AL cardiac amyloidosis, with approximately 8% reaching Perugini grade 3 [18,19]. Our patient exhibited grade 3 uptake with a heart-to-contralateral lung ratio of 2.60; however, the concurrent detection of a lambda-restricted monoclonal protein means that the non-biopsy ATTR criterion was not met, and non-invasive imaging alone cannot reliably distinguish ATTR from AL cardiac amyloidosis in this setting. When high-grade bone-tracer uptake coexists with a detectable monoclonal protein, current guidelines recommend amyloid typing by histological or proteomic analysis of an endomyocardial biopsy to exclude concomitant ATTR and AL disease [4,15,20]. The diagnostic hazard illustrated by this case arises precisely when hematologic screening is omitted or deferred. It must be emphasized that tafamidis should not be initiated in any patient with high-grade bone-tracer uptake who has an abnormal serum free light chain ratio or positive immunofixation until AL amyloidosis has been formally excluded - a requirement mandated by current guidelines regardless of the scintigraphic grade [4,15,20]. In this case, proceeding without that exclusion would have exposed a patient with advanced AL amyloidosis and multiple myeloma to ATTR-directed therapy while delaying urgent plasma cell-directed treatment. The case therefore reaffirms a critical principle: complete hematologic screening, including serum and urine immunofixation and free light chain quantification, must be obtained before any scintigraphic-based diagnosis of ATTR is accepted [4,15,20].

From a hematologic standpoint, the magnitude of lambda elevation (more than 1,270-fold above reference) and coexistent multiple myeloma place this patient at the most aggressive end of the AL spectrum [2]. The Mayo 2004 stage IIIb disease carries a historical median survival of under six months and mortality above 30% in the first month of therapy [13,14]. The Mexican consensus statement emphasizes that quantitative free light chains and serum/urine immunofixation must be obtained as first-line studies whenever cardiac amyloidosis is suspected [11]. In an era of disease-modifying therapies for both AL and ATTR, early subtype identification is now an immediate determinant of survival [2,15].

Limitations

Definitive amyloid fibril typing by mass spectrometry-based proteomics or immunoelectron microscopy was not performed; the magnitude of serum lambda elevation and clonal plasma cell infiltration provides unequivocal evidence of an underlying lambda-restricted dyscrasia. TTR gene sequencing was not performed to formally exclude a hereditary variant. Serum immunofixation was not available; quantitative free light chains and 24-hour urine immunofixation with positive Bence Jones proteinuria provided alternative evidence with diagnostic sensitivity exceeding 95%. Cardiac magnetic resonance was not feasible given hemodynamic instability and recurrent pleural drainage requirements.

Additionally, endomyocardial biopsy with histological or proteomic amyloid fibril typing was not performed due to institutional unavailability and hemodynamic instability; therefore, concomitant ATTR amyloidosis cannot be formally excluded, in keeping with current guideline recommendations for cases presenting with grade 3 bone-tracer uptake and a detectable monoclonal protein [4,15].

## Conclusions

Cardiac amyloidosis has shifted from a clinical rarity to a mandatory differential within the restrictive cardiomyopathy phenotype. Refractory unilateral pleural effusion in a patient with diuretic-resistant heart failure should prompt systematic evaluation for amyloid infiltration, even before overt biventricular hypertrophy is apparent. Equally, PYP scintigraphy must never be interpreted as etiologically definitive in isolation: prior or concurrent exclusion of plasma cell dyscrasia by serum free light chains and serum/urine immunofixation remains the cornerstone of accurate amyloid subtyping, since grade 2 or 3 bone-tracer uptake can occur in the setting of lambda-restricted plasma cell dyscrasia highly consistent with AL cardiac amyloidosis, and does not by itself confirm ATTR amyloidosis. Integrating electrocardiography, multimodal imaging, and hematologic biomarkers into a single coherent diagnostic loop is essential because delayed subtype identification carries direct survival consequences in the era of disease-modifying therapy for both AL and ATTR amyloidosis.
